# Endurance Exercise Attenuates Plasma Level of Neurofilament Light Chain and Prevents Body Weight Loss in a Rat Model of Tauopathy

**DOI:** 10.33549/physiolres.935665

**Published:** 2025-12-01

**Authors:** Marian HORVATH, Michaela SKRABANOVA, Martin CENTE, Jozef HANES, Anton KEBIS, Stefan ZORAD, Peter FILIPCIK

**Affiliations:** 1 Institute of Neuroimmunology, Slovak Academy of Sciences, Bratislava, Slovakia; 2 Slovak Medical University, Bratislava, Slovakia; 3 Institute of Experimental Endocrinology, Biomedical Research Center, Slovak Academy of Sciences, Bratislava, Slovakia

**Keywords:** Physical activity, Weight loss, Tauopathy, Neurofilament light chain

## Abstract

Although its effectiveness and underlying mechanisms are not fully understood, regular physical exercise (PE) is a potential low-cost strategy to prevent or delay neurodegeneration. Plasma neurofilament light chain (NfL), an established biomarker of axonal damage, helps monitor the progression of neurodegenerative disease. Here, we tested whether moderate-intensity endurance exercise modulates body weight trajectories in a rat model of tauopathy expressing human truncated tau protein (WKY72) and how it is associated with plasma NfL. Three months old Wistar Kyoto (WKY) and tauopathic WKY72 rats underwent 10 weeks long treadmill training regimen (30 min/day, 5 days/week). NfL was quantified in plasma of experimental animals collected before the experiment, then after 4 and 8 weeks long training. Sedentary controls were tested in parallel. Body weights were recorded at the same intervals and additionally two weeks later. We found that sedentary WKY72 rats displayed a significant 8.9-fold increase in NfL, while trained WKY72 animals showed only a 3.8-fold increase (both p < 0.0001). In WKY rats, exercise paradoxically led to a modest yet significant increase in NfL (2.9-fold, p < 0.001). Moreover, PE prevented the late-stage weight loss observed in sedentary tauopathic rats. In conclusion, moderate-intensity endurance exercise attenuates plasma level of NfL in tauopathic rats, indicating the potential of exercise as a disease-modifying intervention. Our findings establish a framework for further mechanistic exploration of links between PE and neuroprotective processes.

## Introduction

Neurodegeneration, dementia and obesity are converging global health emergencies, yet up to 45 % of dementia risk is attributable to modifiable factors such as mid-life obesity and physical inactivity [1–3]. PE not only limits weight gain, but also improves cognition and reduces the incidence of dementia, whereas involuntary weight loss in later life often heralds neurodegeneration, preceding the diagnosis by up to a decade [4]. Unintentional body weight loss is associated with majority of neurodegenerative diseases, and significantly contributes to the morbidity and mortality [5]. Due to the complex pathophysiology, we still do not understand details of this phenomenon [6]. NfL is a blood biomarker that sensitively reflects axonal damage in neurodege-nerative disorders [7], and could detect early neuronal death related to wasting or, conversely, exercise-induced neuroprotection. In order to examine these relationships, we used a well-characterised Wistar-Kyoto rat model (WKY72), which develops progressive tauopathy associated with significant body weight loss in parallel with the expression of pathological form of human tau protein [2,8,9]. In the present study we hypothesized, that physical exercise initiated in the pre-symptomatic phase of neurodegeneration delays the first symptoms (e.g. body weight loss), and this effect can be monitored using NFL, a marker of axonal degeneration.

**Fig. 1:**
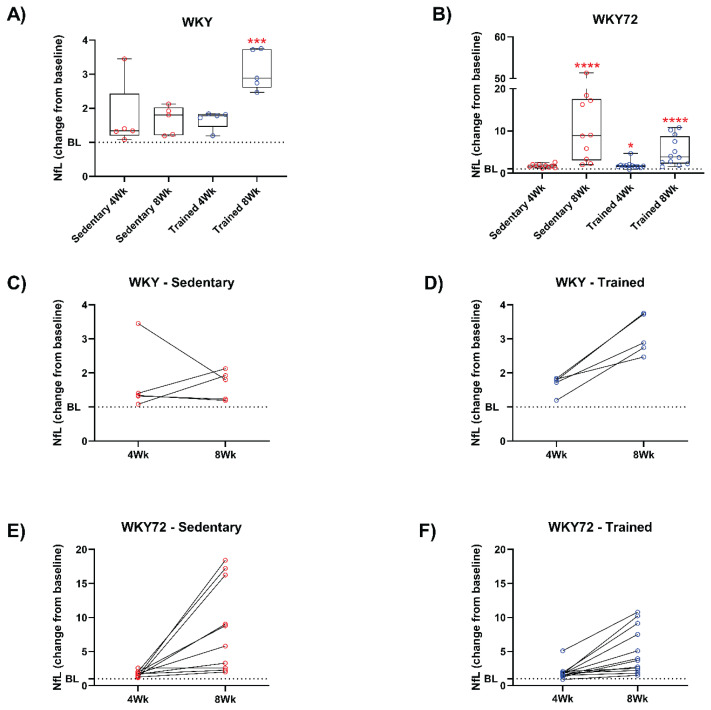
Longitudinal levels of NfL in plasma of control (WKY) and tauopathic (WKY72) rats. Comparison of the NfL change from baselines between two time points for control (**A**) (Sedentary n = 5, Trained n = 5), and tauopathic WKY72 (**B**) (Sedentary n = 10, Trained n = 12) rats. The remaining panels represent longitudinal dynamics of the NfL change from baseline values in individual animals – sedentary (**C**) (n = 5), trained (**D**) (n = 5) control rats, and sedentary (**E**) (n = 10) and trained (**F**) (n = 12) tauopathic WKY72 rats. Boxplots indicate the interquartile range and median, whiskers minimum and maximum, and circles denote individual values. Lines in panels C-F connect values of the same subject. Statistical comparisons with baseline (BL) are denoted in red. * p ≤ 0.0332, *** p ≤ 0.0002 **** p < 0.0001. Within-genotype baseline values were comparable; genotype baselines differed.

Male rats aged 3 months were randomised to a 10 weeks long moderate-intensity endurance training protocol compared to sedentary housing. We quantified plasma NfL at baseline and after 4 and 8 weeks of PE, along with regular body weight measurements, to test whether the physical exercise can prevent weight loss and how it relates to circulating NfL levels during the progression of tauopathy. Male transgenic WKY72 rats (expressing human truncated tau protein under the control of the Thy-1 promoter) and their wild type Wistar-Kyoto non-transgenic littermates were housed in standard laboratory conditions with access to water and food *ad libitum*. All animal experiments were carried out according to institutional animal care guidelines that comply with international standards and were approved by the State Veterinary and Food Committee of the Slovak Republic (Ro-3557/2022-220). The animals were randomly sorted into experimental groups: sedentary and trained. Moderate-intensity running exercise training was conducted for 10 weeks, 5 times a week for 30 minutes, at 16 m/min with an incline 0°. A low-intensity electrical grid delivering the shock (0.7 mA), which did not last more than two seconds was available as a motivational cue and used minimally and uniformly after habituation. No signs of injury or persistent vocalisation were observed. Sedentary rats were placed on the turned off treadmill for the equivalent time. The weight of the animals was measured weekly, however food intake was not recorded. Blood was collected from the lateral tail vein at the beginning of the experiment (pre-training time point) and then after 4 and 8 weeks long endurance training, and plasma was extracted according to the standard protocol (10 μL of 0.1 M EDTA per 200–400 μL whole blood) and frozen at -80 °C until use. Plasma NfL levels were determined using a fully automated digital immunoassay instrument Simoa (single-molecule array) HD-1 Analyzer and the Simoa^TM^ NF-Light Advantage Kit. The proprietary Simoa^TM^ HD-1 instrument software was used to calculate plasma concentrations. Samples were blinded prior analysis and measured in duplicates or monoplicates in case of insufficient amount of material. To evaluate the differences in plasma NfL between sedentary and trained rats, the change from baseline was calculated individually for each animal (at the start of the experiment = pre-training value, at 4 weeks of training and at 8 weeks of training). Change-from-baseline was computed as FC_t = NfL_t / NfL basal value (pre-training value). The baseline value (Fig. 1) was arbitrarily set to 1. Statistical analysis was performed using the non-parametric Kruskal-Wallis test with Dunn’s correction, considering the p < 0.05 significant, in GraphPad Prism 10.5.0 software.

**Fig. 2:**
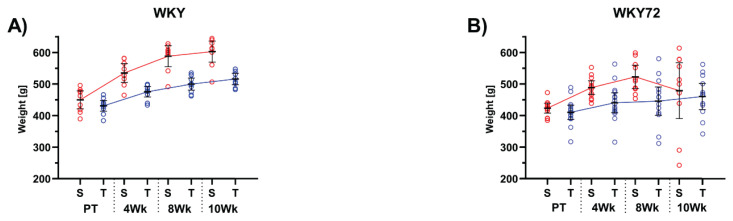
Temporal changes in body weight of sedentary (n = 9) and trained (n = 10) control WKY rats **(A)**, and sedentary (n = 12), and trained (n = 15) tauopathic WKY72 rats **(B)**. Means are connected with trendlines and denoted with 95% confidence intervals (horizontal line and whiskers, respectively). Abbreviations on x axis: S=sedentary animals, T=trained animals, PT=Pre-Trained values, 4Wk=4 weeks of training, 8Wk=8 weeks of training and 10Wk=10 weeks of training.

In the present study, we show that neurode-generation induced by expression of truncated tau protein, as published previously [9], is associated with increased plasma level of NfL in an animal model of tauopathy. According to our previous experiments, the truncated tau protein induces aggressive neuropathology. We found that peripheral NfL levels in sedentary controls, as well as in trained animals, increase longitudinally during aging. NfL levels were significantly higher already in 3 months old tauopathic animals, although they do not show obvious neurodegenerative symptoms. Sedentary tauopathic animals have almost 40-fold higher plasma NfL levels compared to sedentary controls. At 4 months of age, the difference is approximately the same (42-fold higher plasma NfL levels in WKY72 compared to control). However, at 5 months of age, we observed a significant 280-fold increase in plasma NfL in tauopathic rats compared to controls. This NfL profile may indicate that neuropathology in tauopathic animals is significantly accelerated after the 4 months of age. Considering these biomarker data, we selected pre-symptomatic animals at the age of 3 months, to study the effect of PE on the pathogenesis of tauopathy. PE protocol was therefore initiated at 3M of age and lasted for 10 weeks. Next, we show that physical endurance exercise is associated with NfL levels in both healthy controls and tauopathic animals. In control animals, we observed that the change from baseline values increased modestly in both sedentary and 4-week-trained groups. Interestingly, 8 weeks long PE led to 2.9-fold increase in NfL over baseline (p = 0.0005; Fig. 1A). In tauopathic animals, we observed a small increase in NfL in both sedentary and 4-week-trained animals, which was significantly different from baseline in the trained cohort (1.69-fold, p = 0.046). Eight weeks long training led to a 3.8-fold increase in NfL in transgenic animals, while in sedentary transgenic rats, we observed a dramatic 8.9-fold increase (both p < 0.0001) (Fig. 1B). Longitudinal NfL profiles reveal a change in baseline values in individual animals, suggesting that the change in NfL is associated with endurance training in both control and tauopathic animals. Although, in sedentary control rats we did not observe a consistently higher rate of the NfL change (Fig 1C); a distinct, albeit unexpected increase was observed in trained controls (Fig. 1D). Interestingly, sedentary tauopathic animals exhibited a strong 18.4-fold increase in NfL between the timepoints 4Wk and 8Wk (Fig. 1E), while the trained transgenic animals revealed only a 10.8-fold increase (Fig. 1F). These data suggest that even in the case of relatively aggressive neuropathology, which is typical for the tauopathic model in our study, PE may exert a significantly beneficial effect on the progression of neurodegeneration. This presumption needs to be confirmed by further behavioural studies and detailed molecular analyses of brain tissue. Along with the slower rate of increase in plasma NfL in the trained group of tauopathic animals, we observed that endurance training leads to the prevention of weight loss, a typical symptom of tauopathy at this age. Since weight loss is a common feature of later stages of neurodegeneration, we consider our observation to be an interesting and important phenomenon. As shown in Figure 2, the endurance training regimen resulted in a continuous slowdown in weight gain in control animals (Fig. 2A,B). Both WKY groups (sedentary and trained) had comparable mean weight values at the start of the experiment (Sedentary 450 ± 12 g, n = 9; Trained 431 ± 7 g, n = 10). However, the weight of sedentary animals increased by approximately 34 %, while the trained animals only gained approximately 20 %. The baseline mean weight values of the tauopathic WKY72 rats were comparable to the WKY controls (Sedentary 423 ± 7 g, n = 12; Trained 411 ± 11 g, n = 15; Fig. 2C,D). The weight gain of sedentary WKY72 rats was initially similar to that of controls, but 8 weeks after the start of the experiment (corresponding to the age of 5M) we observed a decrease in body weight, ending at 479 ± 39 g. In contrast, endurance exercise maintained the increasing trend of the body weight curve without any signs of body weight decrease in tauopathic animals (461 ± 19 g). As a result, sedentary WKY72 rats were about 20 % lighter at week 10 than the sedentary control group, while the difference between trained cohorts (WKY72 vs. WKY) was only 11 %.

Our study revealed that 10 weeks long moderate-intensity treadmill running was able to prevent late-stage body weight loss, an important symptom reflecting the truncated tau-induced neurodegeneration, in tauopathic animals. The increase in plasma NfL, a neurodegeneration marker associated with axonal damage in this tauopathy, was significantly attenuated after the 8 weeks long training. It is not excluded that trained animals have different metabolic pathways or metabolic rates associated with the production or degradation of NfL than sedentary ones. Also, the use of an aversive stimulus could affect stress biology and energy balance. However, since the trained wild-type cohort did not lose weight, we do not consider grid exposure or stress markers as factors modifying the results. An intervention that modulates weight loss and counterbalances the increased NfL could be clinically meaningful, as both of these phenomena predict a faster functional decline in people with Alzheimer’s disease (AD) and frontotemporal dementia (FTD) [10,11]. Hence, endurance exercise might be well-positioned as a disease-modifying rather than merely symptom-modifying strategy in early tauopathy. It was previously published that the loss of body weight in sedentary tauopathic rats is associated with the expression of the pathogenic form of tau protein [2]. Interestingly, unlike the voluntary wheel-running study in rTg4510 mice that failed to prevent wasting [12], we observed prevention of body weight loss after PE in our model. The small yet significant increase in NfL in trained control animals is in direct parallel with the transient elevations reported after very strenuous bouts after high-intensity interval training (HIIT) [13], which may even reflect axonal remodelling rather than injury. Forced treadmill exercise is already known to attenuate tau pathology in other tauopathic models [14], and our data extend the knowledge on the homeostasis of body weight and circulating NfL levels. Blood NfL level, as a marker of neuroaxonal damage, is linked to both the onset of symptoms and brain atrophy in tauopathy [15]. Our results indicate that they may be modified by physical exercise, which is also supported by evidence obtained in other neurological disorders [16]. Therefore, longitudinal NfL measurement could serve as an early read-out in lifestyle-based randomised controlled trials. Finally, the divergent body weight trajectories we observed in sedentary rats experiencing weight loss and trained rats maintaining relatively stable body weight reflect the late-stage wasting associated with pre-symptomatic dementia [17], reinforcing the idea that aerobic exercise in early-life may delay the onset of symptoms, even when neurodegeneration is genetically determined.

The strengths of our study include the moderate training dose, serial plasma NfL sampling, and concurrent weight monitoring. Nevertheless, we acknowledge the small sample size, male-only cohorts, mild foot-shock motivation, and absence of direct strength measurements as limitations of this exploratory study. In conclusion, we found that our experimental model is suitable for detailed investigation of molecular mechanisms that link physiological responses induced by physical exercise with neuroprotective processes. Further research may lead to a better understanding of the role of physical exercise in preventing or delaying neurodegeneration.
